# First‐time red blood cell transfusions in the emergency department: A deficiency in micronutrient testing

**DOI:** 10.1111/tme.70068

**Published:** 2026-02-11

**Authors:** Andrea Coetzee, Karryn Brown, Jessica Opie, Jody Rusch, Annemarie Kropman, Vernon Louw

**Affiliations:** ^1^ Division of Clinical Haematology, Department of Medicine University of Cape Town Cape Town South Africa; ^2^ Division of Haematology, Department of Pathology University of Cape Town and National Health Laboratory Service Cape Town South Africa; ^3^ Division of Chemical Pathology, Department of Pathology University of Cape Town and National Health Laboratory Service Cape Town South Africa; ^4^ Division of Emergency Unit, Department of Medicine University of Cape Town Cape Town South Africa

**Keywords:** Anaemia, emergency department, ferritin, folic acid, iron deficiency, micronutrient testing, micronutrients, nutritional assessment, red blood cell transfusion, vitamin B 12

## Abstract

**Objectives:**

To evaluate laboratory investigations for micronutrient deficiencies as the cause of anaemia prior to first‐time red blood cell transfusions in the medical emergency department (ED) of a large academic hospital.

**Background:**

The ED is often the first point of contact for acutely ill anaemic patients, yet patient blood management principles are underutilised. Because micronutrient deficiencies are common, treatable causes of anaemia, they should be screened for to guide targeted treatment and reduce unnecessary transfusions.

**Methods:**

We conducted a retrospective cross‐sectional study of 198 adult patients receiving first‐time transfusions in the medical emergency unit at Groote Schuur Hospital, South Africa, between April and October 2023. Laboratory investigations for iron, vitamin B12, and folate deficiencies were analysed and stratified by pre‐transfusion mean corpuscular volume (MCV). Iron deficiency was evaluated using three locally used criteria. Data about haematinic supplementation were also collected.

**Results:**

Only 29% of patients received a full micronutrient deficiency workup, while 39% had no testing. Iron deficiency was the most frequent abnormality, and up to 30% of these patients had normocytic indices. Iron supplementation rates were high, but many patients without iron deficiency also received iron.

**Conclusion:**

The ED encounter should be used as an opportunity to initiate basic micronutrient deficiency screening for anaemia, regardless of MCV.

## 1 INTRODUCTION

Anaemia is a common clinical condition that affects 1.92 billion people worldwide.^1^ In South Africa, community rates of anaemia reached 31% in women and 17% in men over 15 years,^2^, ^3^ with an even greater burden among people living with HIV, where anaemia affects up to 72%.^4^ It is associated with prolonged hospital stays, higher mortality rates, and reduced quality of life.^5^, ^6^ Iron deficiency remains the leading global cause of anaemia in populations across all income levels.^1^, ^7^ Omitting aetiology‐specific diagnoses and treatment may impact patient morbidity and mortality, and place additional strain on already restricted blood supplies in lower‐ and middle‐income settings.^8^ The early diagnosis and treatment of patients whose anaemia is caused by micronutrient deficiencies will mitigate transfusion‐associated risks, as haematinics are safer and more cost‐effective alternatives.^9^

The emergency department (ED) is often the first point of contact for acutely ill anaemic patients. Anaemia investigations in time‐pressured EDs are often suboptimal, as clinicians may view these workups as beyond their scope. Beverina and Brando (2019) found that among ED patients with microcytic anaemia who were transfused, only 22% had their iron status tested.^10^ The 2023 study by O'Mahony et al. of 1327 patients in a rural region of South Africa supported the need to assess iron status in all patients with anaemia, because of the significant prevalence of iron deficiency (26.5%). The authors recommended testing for iron, folate, and vitamin B12 deficiencies in all anaemic patients, regardless of the mean corpuscular volume (MCV). Less than half of patients with iron deficiency anaemia presented with microcytosis.^3^ Screening for micronutrient deficiencies as the cause for anaemia forms part of the first pillar of patient blood management (PBM): optimising red blood cell (RBC) mass using haematinics.^11^

While PBM is well‐established in surgical and inpatient settings, its application in the ED is not common. The aim of our study was to investigate first‐time blood transfusions in the ED, as this information can serve as a useful benchmark for the implementation of PBM. The study could help to identify inappropriate blood product use in the ED and could demonstrate whether anaemia is fully investigated prior to making transfusion decisions.

## 2 METHODS

This retrospective, cross‐sectional observational study was conducted at Groote Schuur Hospital, a public sector academic institution, located in Cape Town, South Africa. The study included all adult patients who received a RBC transfusion for the first time in the medical emergency unit between April and October 2023. At Groote Schuur Hospital, the ED comprises three clinical areas: the medical emergency unit, the gynaecological emergency unit, and the trauma unit. The medical emergency unit (the focus of our study) manages undifferentiated medical and surgical presentations, while major trauma is seen exclusively in the trauma unit. A list of patients was extracted from the Western Cape Blood Service database, which includes all previous transfusion episodes in the province. Of 432 transfusion episodes, 234 patients were excluded due to prior transfusions, age under 18 years, absence of a pre‐transfusion haemoglobin, or polytrauma. The final sample included 198 adult patients. Laboratory data from the National Health Laboratory Service (NHLS) Trakcare Lab results system were reviewed for a full blood count (FBC), serum ferritin (SF), transferrin saturation (TSAT), vitamin B12, serum folate, C‐reactive protein (CRP), and available HIV serology. Patient records were reviewed for transfusion indications and haematinic supplementation. Data were captured in a secure REDCap online database. Since this was classified as secondary use of routine clinical data, a waiver of informed consent was approved.

### 2.1 Laboratory results

A full laboratory anaemia workup for micronutrient deficiencies was defined as the testing of SF, TSAT, vitamin B12, and serum folate levels. A partial workup included testing one or more, but not all, of these parameters. A minimally acceptable workup was assessed per MCV category.^12^, ^13^ Patients with an MCV <80 fL required at least an SF, patients with an MCV >100 fL required at least a vitamin B12 and serum folate level, and patients with an MCV 80–100 fL required SF, vitamin B12, and serum folate levels.

Iron deficiency was defined using three locally used SF‐based criteria: (1) SF <13 μg/L, reflecting the lower limit of the NHLS laboratory reference interval;^14^ (2) SF <30 μg/L, a widely supported clinical threshold with improved sensitivity^15^, ^16^; and (3) SF <80 μg/L in isolation, or an SF 80–200 μg/L combined with a CRP >5 mg/L and TSAT <13%, a definition developed by our group that accounts for inflammation and malignancy.^17^ Vitamin B12 deficiency was defined as a serum level <108 pmol/L, in keeping with one of the local laboratory reference intervals. A serum folate level <7 nmol/L was used as the cut‐off for folate deficiency in this study.^18^

### 2.2 Data analysis

Data were analysed using STATA version 18 (Stata Corporation, College Station, Texas, United States). Categorical variables were described as frequencies and percentages. Numerical data were described by medians and the 25th and 75th percentile as data were non‐parametrically distributed. Two groups of continuous variables were compared using the Mann–Whitney *U* test. Pearson's Chi‐squared test or Fisher's exact test (*n* < 5) was used to compare categorical variables. For all analyses, a *p*‐value <0.05 was considered statistically significant.

## 3 RESULTS

### 3.1 Patient characteristics

The final sample included 198 patients, which had a near‐equal sex distribution and a median age of 56 years (40, 70), as seen in Table 1. Among all 198 patients, 19% had positive HIV serology results, 56% had negative results, and 25% had no results available. The median pre‐transfusion haemoglobin (Hb) was 6.0 g/dL (5.1, 7.4).

**TABLE 1:** Baseline characteristics of patients receiving a first‐time red blood cell transfusion in the emergency department between April and October 2023.

		Frequency (%) or Median (P25, P75)			
Variable	Category	Total cohort (*N* = 198)	Patients receiving full workup^a^ (*N* = 56)	Patients not receiving full workup^b^ (*N* = 142)	*p*‐value
Sex	Male	95 (48)	29 (52)	66 (46)	0.501
	Female	103 (52)	27 (48)	76 (54)	
HIV status	Positive	37 (19)	12 (21)	25 (18)	0.496
	Negative	111 (56)	33 (59)	78 (55)	
	Unknown	50 (25)	11 (20)	39 (27)	
Age (years)	Overall	56 (40, 70)	54 (37, 66)	57 (41, 70)	0.368
	18–29	17 (9)	6 (11)	11 (8)	0.823
	30–49	67 (34)	18 (32)	49 (35)	
	50–69	66 (33)	20 (36)	46 (32)	
	70+	48 (24)	12 (21)	36 (25)	
Timing of workup (*n* = 120)	Before or on day of transfusion^c^	110 (92)	53 (95)	57 (89)	0.334
	After transfusion^d^	10 (8)	3 (5)	7 (11)	
MCV (fL)	Overall	84 (76, 92)	82 (75, 94)	84 (76, 91)	0.818
	<80	76 (39)	21 (38)	55 (39)	0.156
	80–100	111 (56)	29 (52)	82 (58)	
	>100	11 (6)	6 (11)	5 (4)	

Abbreviations: MCV, mean corpuscular volume; P25, 25th percentile; P75, 75th percentile.

^a^ Serum ferritin, transferrin saturation, vitamin B12, and serum folate levels performed.

^b^ Includes 64 patients that received a partial workup (one or more parameters) and 78 that had no workup.

^c^ Performed within two weeks before transfusion.

^d^ Performed within two weeks after transfusion.

### 3.2 Transfusion indications

A review of patient records provided clinical information regarding the reason for ED admission and subsequent transfusion and micronutrient testing. Overall, 38% of the study population presented with bleeding, most commonly gastrointestinal (24%), followed by genitourinary (8%), respiratory (4%), and vascular causes such as abdominal aortic aneurysm and aortic dissection (3%). Among patients without bleeding, anaemia of inflammation accounted for 23% of transfusions, micronutrient deficiency anaemia for 13%, and haematological disorders for 13%. A smaller proportion of patients (7%) were transfused for pre‐operative optimization and 5% for other or unclear indications.

### 3.3 Micronutrient deficiency screening

Of 198 patients, 56 (29%) received a full micronutrient deficiency workup, while 78 (39%) had no testing pre‐ or post‐transfusion. Among the 120 patients who received a full or partial workup, 92% were tested pre‐transfusion. No differences in age, sex, or HIV status were found between those who received a full workup and those who did not. Among patients with microcytosis (MCV <80 fL), 48/76 (63%) had an SF test performed. In the group with macrocytosis (MCV >100 fL), 8/11 (73%) had vitamin B12 and folate testing performed, and in the group with normocytosis (MCV 80–100 fL), 34/111 (31%) had SF, vitamin B12, and folate testing performed.

### 3.4 Frequency of micronutrient deficiencies

Table 2 outlines the frequency of each micronutrient deficiency. Using an SF cut‐off of <13 μg/L identified 15 of 110 patients as iron deficient; however, this threshold would have missed an additional 14 iron‐deficient patients compared with an SF cut‐off of <30 μg/L, which identified 29 of 110 patients as iron deficient. The third method, incorporating a higher SF cut‐off, a TSAT and CRP, identified 40 of 102 patients as iron deficient. One of 72 patients tested had folate deficiency, which occurred in a case of microcytic anaemia, but no iron testing was performed to assess for concurrent iron deficiency. Of 88 patients tested, there were only two patients with vitamin B12 deficiency: one with microcytosis and concurrent iron deficiency, and the other with macrocytosis. Both patients with vitamin B12 deficiency had supplementation prescribed, whereas the patient with folate deficiency did not.

**TABLE 2:** Micronutrient deficiencies overall, and per mean corpuscular volume (MCV) category, using different cut‐off values for iron deficiency.

		MCV (fL)			
		<80	80–100	>100	Total
Micronutrient deficiency	Iron deficiency^a^	15/48	0/56	0/6	15/110 (14%)
	Iron deficiency^b^	24/48	5/56	0/6	29/110 (26%)
	Iron deficiency^c^	28/45	12/51	0/6	40/102 (39%)
	Folate deficiency	1/26	0/38	0/8	1/72 (1%)
	Vitamin B12 deficiency	1/31	0/49	1/8	2/88 (2%)

Abbreviation: MCV, mean corpuscular volume.

^a^ Iron deficiency defined as serum ferritin (SF) < 13 μg/L.

^b^ Iron deficiency defined as SF <30 μg/L.

^c^ Iron deficiency defined as SF <80 or 80–200 μg/L plus C‐reactive protein (CRP) >5 mg/L plus transferrin saturation (TSAT) <13%.

### 3.5 Iron supplementation

Iron supplementation data were available for 181 patients (see Table 3). Of these, 63 (35%) received iron. Of the 63, 17 (9%) received intravenous iron, 30 (17%) received oral iron, and 16 (9%) received both oral and intravenous iron. Iron supplementation was common among patients with iron deficiency: 14 of 15 patients with an SF <13 μg/L, 24 of 29 patients with an SF <30 μg/L, and 31 of 40 patients with either an SF <80 μg/L or 80–200 μg/L with a CRP >5 mg/L and a TSAT <13%. Some patients received iron supplementation despite not meeting any definition of iron deficiency, including in the group with higher SF values. There were 17 patients who received iron despite not having SF testing performed.

**TABLE 3:** Iron supplementation rates across different definitions for iron deficiency.

Different cut‐off values for iron deficiency	N evaluable	*n*	Patients receiving iron
SF <13 μg/L	110	15	14
SF ≥13 μg/L		95	32
SF <30 μg/L	110	29	24
SF ≥30 μg/L		81	22
SF <80 or 80–200 μg/L & CRP >5 mg/L & TSAT <13%	102	40	31
SF > 200 or SF 80–200 μg/L & CRP ≤5 mg/L or SF 80–200 μg/L & CRP >5 mg/L & TSAT ≥13%		62	13
SF untested	198	88	17

Abbreviations: CRP, C‐reactive protein; SF, serum ferritin; TSAT, transferrin saturation.

## 4 DISCUSSION

In this study of first‐time ED transfusions, less than one‐third of patients received a full micronutrient deficiency workup, while over one‐third had no investigations performed in the two weeks before or after transfusion. Stratification by MCV to tailor the workup to the minimally acceptable standards for each MCV category was associated with a higher rate of appropriate workups in the microcytic and macrocytic groups (63% and 73%, respectively). The normocytic group had the lowest rate of appropriate workups at 31%, suggesting clinicians may overlook micronutrient deficiency screening when MCV values are normal.

Our results confirmed that in the ED, anaemia often does not receive appropriate laboratory investigation, a finding consistent with previous studies.^10^, ^19^ ED clinicians may also view testing for micronutrient deficiencies as outside their role, as shown by Berard et al., who found that 67% of surveyed paediatric ED physicians did not consider diagnosing iron deficiency their responsibility.^20^ The same trend has been described in hospitalised inpatients. In a recent study by Carrier et al. of hospitalised patients with anaemia, 37% of patients had an insufficient laboratory anaemia workup and less than half had iron studies performed. Patients with unidentified anaemia, defined as having no laboratory investigations performed or no identified cause, were more likely to be middle‐aged and to have a higher nadir Hb level.^21^ No differences were found between patient subgroups in our study. The median pre‐transfusion Hb in our study (6.0 g/dL) fell within the range of severe anaemia and below restrictive transfusion thresholds (typically 7–8 g/dL).^22^ Although the relationship between individual Hb concentrations and testing was not evaluated, a large proportion of patients presented with severe anaemia, warranting investigation.

Diagnosing iron deficiency using SF can be challenging due to inconsistent use of cut‐off values and reference intervals. The use of lower SF thresholds may underestimate iron deficiency, particularly in women, as the lower SF thresholds are based on outdated consensus meetings rather than robust evidence.^23^, ^24^ Truong and co‐authors noted that SF reference intervals considerably lower than 30 μg/L are commonly used, despite strong evidence indicating that values below this threshold have an unacceptably low sensitivity and an unnecessarily high specificity for diagnosing iron deficiency.^15^, ^24^ In our setting, the lower limit of the local SF reference interval (<13 μg/L) identified 14% of patients with iron deficiency. Raising the cut‐off to <30 μg/L doubled the detection and identified cases with a normal MCV, often seen in early iron deficiency. The highest‐yield approach recently reported by Richardson et al.^17^ incorporates an SF cut‐off of <80 μg/L or an SF of 80–200 μg/L with a CRP >5 mg/L and TSAT <13%. These criteria have a sensitivity of 78% and specificity of 92%, and in our study, led to a diagnosis of iron deficiency in 39% of patients, including 12 patients with a normal MCV. It should be noted that although bone marrow iron stores were used as the benchmark, the population studied consisted largely of patients with infections and/or haematological malignancies and therefore may not be generalisable to all patients. The variation in the yield of positive test results using these different cut‐off levels highlights the need for standardised cut‐off values. No patients with iron deficiency had macrocytic anaemia, suggesting that in resource‐limited settings, iron studies might not be needed when patients present with macrocytic anaemia.

Our study found low rates of vitamin B12 and folate deficiencies, with two of the three cases surprisingly having microcytosis. One had folate deficiency with unknown iron status (untested), and the other had vitamin B12 deficiency and concurrent iron deficiency. This may be due to low testing rates in the normocytic and macrocytic subgroups. We would like to argue that relying solely on the traditional MCV‐based approach to guide anaemia workups might miss early or overlapping micronutrient deficiencies, particularly in normocytic and microcytic anaemia. While pre‐transfusion testing is ideal, evidence suggests that common biochemical parameters (SF, TSAT, serum folate, and vitamin B12) may remain reliable to diagnose the presence of an underlying micronutrient deficiency up to 14 days post‐transfusion.^25^, ^26^

Iron supplementation rates among iron‐deficient patients were high. According to the current lower limit of our local laboratory reference interval (SF <13 μg/L), the patients were treated well (14 of 15 patients). This suggests that iron deficiency, once diagnosed, is appropriately treated in the ED. Notably, many patients received supplementation despite not meeting any of the three definitions for iron deficiency, and some received iron without having even an SF performed. While the dosage was not directly measured, common practice in our ED is to administer a total dose infusion of 1 gram low molecular weight iron dextran (CosmoFer®). This reflects typical practice in time‐pressured ED settings where physicians do not always perform individualised iron dose calculations, potentially exposing patients to higher doses of iron in the absence of iron deficiency. We recommend testing before initiating treatment, as iron deficiency is only one of many causes of anaemia.

This study had several limitations. Urgent transfusions in acutely bleeding patients may have led to omission of full workups. Acute anaemia with haemodynamic instability should be managed with resuscitation and transfusion, as reflected in the Spradbrow et al. algorithm for evaluating transfusion appropriateness in iron deficiency anaemia.^19^ Nevertheless, these patients would usually need a crossmatch anyway and we would recommend that blood is drawn for micronutrient testing at the same time for appropriate follow‐up and action after resuscitation. We included bleeding patients (38% of transfusion indications) to ensure that individuals with chronic iron deficiency resulting from gastrointestinal and other bleeding were not overlooked. The tertiary referral setting's high burden of chronic inflammatory conditions may have influenced the interpretation of iron studies. To address this, we evaluated a recently developed algorithm for diagnosing iron deficiency in the context of inflammation and recommend the addition of TSAT and CRP levels to improve iron study interpretation in such patients.^17^ Lastly, we only included patients who were transfused and may have excluded chronically anaemic patients appropriately treated with haematinics rather than transfusion. The results of this research were practice‐informing and were shared with trainees and permanent staff of the ED to ensure adequate feedback on current practices and PBM metrics, inspiring motivation for improvement.

In conclusion, the ED encounter should be viewed as a point of intervention to initiate basic screening for micronutrient deficiencies as the cause for anaemia. Identified deficiencies should prompt further investigation into underlying causes.

## AUTHOR CONTRIBUTIONS

Vernon Louw, Andrea Coetzee, and Annemarie Kropman conceptualised and designed the study, with input from Jessica Opie and Jody Rusch. Andrea Coetzee was responsible for data collection and data management. Karryn Brown performed the data analysis and assisted with data management. Andrea Coetzee drafted the initial manuscript. All authors critically reviewed the manuscript and approved the final version.

## FUNDING INFORMATION

Research reported in this publication was supported by the Fogarty International Center and National Heart, Lung & Blood Institute of the National Institutes of Health under Award Number D43 TW010345.

## CONFLICT OF INTEREST STATEMENT

The authors have no competing interests.

## PATIENT CONSENT

Since this was classified as secondary use of routine clinical data, a waiver of informed consent was approved.

## Data Availability

The study data are not publicly available as it contains personal patient information.
